# Endoscopy in Pediatric Eosinophilic Esophagitis

**DOI:** 10.3389/fped.2021.713027

**Published:** 2021-08-24

**Authors:** Nathalie Nguyen, Robert E. Kramer, Calies Menard-Katcher

**Affiliations:** Department of Pediatrics, Digestive Health Institute, Children's Hospital Colorado, University of Colorado School of Medicine, Aurora, CO, United States

**Keywords:** eosinophilic oesophagitis, endoscopy, dilation, pediatric, children, esophagogastroduodenoscopy

## Abstract

Endoscopy and mucosal biopsies are essential to the diagnosis of EoE. Together they either confirm or exclude mucosal eosinophilia and provide a visual inspection of the esophagus that may be consistent with EoE or suggest other underlying etiologies. Endoscopy also plays an important therapeutic role in the management of EoE including the assessment of treatment response and treatment of associated complications including esophageal stricture and food impaction. Assessment of treatment response largely depends on endoscopy and mucosal biopsies although less invasive strategies may eventually provide alternative means to assess mucosal inflammation. Herein we will review current use of endoscopy in EoE, including recently developed technologies and their role in the management of EoE.

## Introduction

During the last two decades, an emerging body of clinical experiences and research studies have identified eosinophilic esophagitis (EoE) as the most common cause of food impaction and a common cause of dysphagia and esophagitis in children and adults. The incidence of EoE ranges from 5 to 10 cases per 100,000 (1) and it has been reported to occur worldwide (1). Eosinophilic esophagitis is a chronic immune antigen-mediated disease characterized by symptoms of esophageal dysfunction and inflammatory changes in esophageal mucosa including >15 eosinophils per high power field on biopsy (2, 3). Endoscopy and mucosal biopsies are essential to the diagnosis of EoE by either confirming or excluding mucosal eosinophilia and providing a visual inspection of the esophagus that may be consistent with EoE or suggest other underlying etiologies (4, 5). Endoscopy also plays an important therapeutic role in the management of EoE including the assessment of treatment response and treatment of associated complications including esophageal stricture and food impaction. Assessment of treatment response largely depends on endoscopy and mucosal biopsies although less invasive strategies may eventually provide alternative means to assess mucosal inflammation. Herein we will review current use of endoscopy in EoE, including recently developed technologies and their role in the management of EoE.

## Role of Endoscopy in Eosinophilic Esophagitis

### Obtaining Mucosal Biopsies

The gold standard for diagnosis of EoE requires mucosal biopsy for histological assessment to evaluate for its characteristic eosinophil predominant inflammation of the esophageal epithelium (defined by >15 eosinophils per high power field) (2). Visual inspection of the esophagus alone is not sufficient as a reliable marker of tissue involvement in EoE (6). Esophageal biopsies should be obtained from multiple locations along the esophageal length including the distal, mid and/or proximal esophagus. Two previous studies in adults and children have suggested that obtaining a total of six biopsies from at least two sites increases the probability of establishing the diagnosis of EoE to over 95% (7, 8). The normal esophagus is devoid of eosinophils and eosinophil enumeration is most often used to describe the severity of inflammation. However, several other histologic features have been described in eosinophilic esophagitis, including basal cell hyperplasia, dilated intercellular spaces, rete-peg elongation, lamina propria fibrosis, eosinophilic microabscesses and eosinophil layering of the surface epithelium and collectively are used in the EoE Histologic Severity Score (EoEHSS) (9, 10). The EoEHSS is a validated histologic measure for EoE that provides a broad assessment of epithelial inflammation beyond eosinophil density.

### Identification of Endoscopic Findings

**Figure 1:**
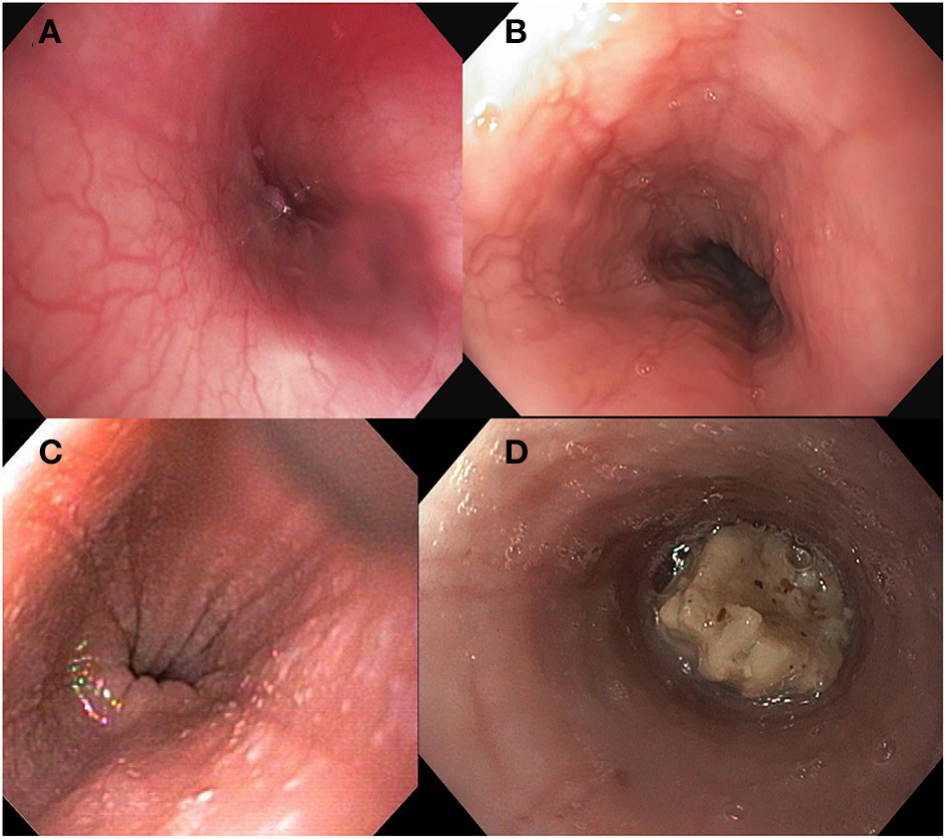
Endoscopic findings in Eosinophilic Esophagitis including **(A)** normal esophageal mucosa **(B)** longitudinal furrows, **(C)** white exudate, and **(D)** food bolus impaction.

Several endoscopic findings are associated with EoE including esophageal edema (decreased vascularity or pallor of the esophageal mucosa), esophageal rings (concentric rings or trachealization in the esophagus), white exudate (white spots or plaques), longitudinal furrows, esophageal strictures, narrow caliber esophagus (reduced caliber of the majority of the esophagus), and crepe paper esophagus (mucosal fragility of the esophagus). See Figure 1. Studies have identified that more than 90% of patients with EoE will have at least one abnormal endoscopic feature of EoE (4). The Endoscopic Reference Score (EREFS Score) is a numerical scoring system that grades both the presence and severity of endoscopic features including edema, rings, exudate, furrows and stricture (4). The EREFS score provides a standard method of assessing the endoscopic appearance of EoE and can assist in identifying patients as having only inflammatory findings (e.g., white plaques, linear furrows, edema) as compared to fibrotic features (e.g., esophageal rings or stricture) (4). Although not a universal conclusion, studies in both adult and pediatric subjects concluded that the EREFS score accurately identified patients with EoE and can be used as an endoscopic outcome measure of response to treatment (5, 11, 12). Biopsies and histologic inflammation remain the primary marker of disease activity but endoscopic appearance provides a practical adjunct assessment of disease activity at diagnosis and of treatment response.

Visual inspection of the esophagus is also helpful in patients to assess for alternate etiologies of esophageal symptoms (candida esophagitis, herpes esophagitis, or erosive esophagitis).

### Assessment of Treatment Response

Treatment of inflammation is important to improving the natural history of disease, preventing complications including food impaction and esophageal stricture (13, 14). Endoscopy with biopsy remains an essential tool in the assessment of treatment response. It has been recognized that patient reported symptoms do not necessarily correlate well with histology, particularly in those treated or partially treated for EoE. Patient reported symptom assessment tools have been developed for both adult and pediatric population which provide a standard means of assessing symptom severity; however there continues to be only a moderate level of association in symptom and histologic response to treatment (15–17). Therefore, tissue assessment has been and continues to be an important tool to assess treatment response. Most studies to date have evaluated response to treatment by endoscopy at 6–16 weeks after initiation of treatment and this time frame, while broad, generally is accepted practice. Results from ongoing and future studies will help us determine what time frame is optimal.

The downside of assessing histologic response to treatment is the increased need for invasive procedures along with associated patient and health care costs. In addition, there has been increasing attention on repeated use of anesthesia in young children, particularly after the US Food and Drug Administration issued a “Drug and Safety Communication” warning that repeated use of anesthetics may affect development of children's brains (https://www.fda.gov/Drugs/DrugSafety/ucm532356.htm). This has motivated the search for a biomarker of tissue inflammation. However, to date no specific serum, blood, breath, or urine biomarkers has been validated to differentiate between active and inactive esophageal eosinophilia. Less invasive means of esophageal sampling without the need for anesthesia have begun to show promise and are described below.

### Therapeutic Endoscopy in EoE

Patients with EoE can have esophageal complications including food impaction and esophageal stricture and endoscopy is an important tool in the management of both.

#### Food Bolus Impaction

Food bolus impaction is often a presenting symptom of EoE and occurs in 33–55% of children and adults with EoE. When a presenting symptom, obtaining esophageal biopsies is recommended during endoscopy for food impaction removal in order to assist in making a timely diagnosis (18, 19). Gastroenterologists, surgeons and otolaryngologists may all be asked to assist in the removal of esophageal food impaction. When located in the proximal esophagus, rigid endoscopy may be considered but flexible endoscopy is more often utilized. Methods to remove food range from using either single or multiple devices, including snare, net retriever, tripod grasper, rat tooth forceps, biopsy forceps, and suction. Suction using a transparent suction cap secured to the end of the endoscope or bander can be effective and may reduce procedure time compared to other pull removal techniques (20). Often, difficult impactions require the use of multiple tools as they are rarely removed as a single piece. Due to the frequent need for multiple passes of the endoscope to fully remove an impaction, use of an overtube should be considered in children large enough to accommodate them to minimize the potential trauma of repeated esophageal intubation. While gentle pressure to “push” the impaction into the stomach can be considered, extreme caution should be exercised as it often unknown if there may be a more distal stricture and longitudinal tearing of the mucosa or perforation may occur.

Timing of this procedure is urgent if there is drooling or other evidence of complete esophageal obstruction that puts the patient at risk for aspiration. For this reason, as well as the likelihood of repeated passes of the endoscope, use of an endotracheal intubation should be strongly considered. With complete obstruction urgent endoscopy (<8 h) should be performed regardless of *nil per os* (NPO) status. If the patient is able to manage their own secretions, removal of the impacted food bolus should nevertheless be performed within 24 h from the onset of symptoms to avoid tissue necrosis and the risk of perforation during the procedure.

#### Esophageal Stricture

Focal stricture or long segment narrowing occur in a subset of children and adults with EoE. Stricture severity is typically characterized as mild, moderate, or severe based on the ability to pass either a standard or pediatric sized endoscope. Strictures may occur with or without presence of esophageal rings. Mild strictures can be detected by endoscopy but diffuse or long strictures often require a high index of suspicion and complete esophageal insufflation. Studies in both children and adults demonstrate that narrowing can be missed in up to 55% of patients if endoscopy alone is used as a diagnostic tool, as compared to barium esophagram and endoscopy together (21, 22). If a patient has solid food dysphagia, performance of a barium esophagram, often with a barium pill, can be helpful in assessing for the presence of luminal narrowing (23). More frequently, EoE related strictures are long segment or diffuse making them more amenable to bougie dilation with either Maloney or wire guided Savary dilators rather than balloon dilation. When focal strictures exist, balloon dilation is a reasonable approach and has the benefit of offering direct visualization during dilation as well as directing all of the force radially. A balloon pull-through technique has also been described for adults in the management of EoE narrowing (24). Complications include bleeding and esophageal perforation however, several studies in adults and children including a systemic review have found these complications to be rare and no more frequent than in esophageal dilations for other underlying etiologies (25, 26). Additionally, a meta-analysis comparing dilation method found no evidence to suggest a significant difference in perforation risk related to dilator type (27). Post-operative chest pain; however, is expected in 15–74% of patients and can be preemptively managed by providing anticipatory guidance and symptomatic pain relief if needed with non-narcotic pain medications (25, 26). With the presence of stricture, longitudinal “rents” in the mucosa are often seen with passage of the endoscope even before dedicated dilation is performed. This is expected and should not be necessarily interpreted as a result of undue trauma or an adverse event. Though the “rule of three” standard dilation practice advises against dilation of more than 3 mm within a single session, single center data in pediatric EoE patients has not shown an association with the final dilator size and risk of perforation, with a mean increase of 4.5 mm per dilation (26).

More than half of patients necessitating dilation will require repeat dilation in their symptom management. In adults, repeat dilation was often needed within a year of initial dilation (26, 28). While dilation can improve dysphagia when used in the appropriate patient, it should not be viewed as an alternative to therapies directed at treating inflammation. When inflammation is controlled patients require fewer dilations to achieve a similar improvement in esophageal diameter (29).

## Emerging Endoscopic and Less Invasive Tools in EoE

### Transnasal Endoscopy

**Figure 2:**
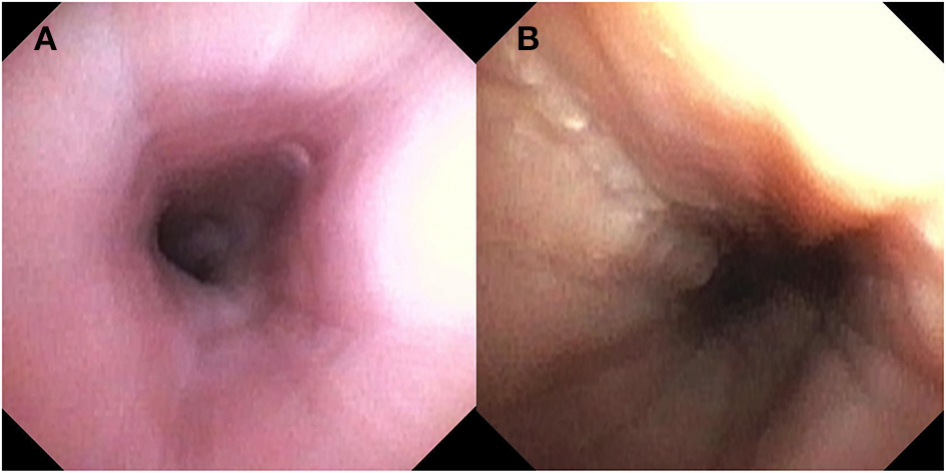
Endoscopic findings with Transnasal Endoscopy including **(A)** normal esophageal mucosa and **(B)** longitudinal furrows and scant white plaques.

Over the last few years, unsedated transnasal endoscopy (TNE) has been performed successfully in children as an alternative to EGD for surveillance of EoE (30, 31). In an outpatient clinic room, children wear video or virtual reality goggles for distraction and TNE is performed using an ultrathin bronchoscope (with an outer diameter of 2.8–4.2 mm). This allows for direct visualization of the esophagus and esophageal biopsies are obtained (31). Endoscopic features such as white plaques are readily visible and, with adequate insufflation, other features such as linear furrows and edema are possible however how a standardized endoscopic score obtained during TNE compares to one during standard endoscopy has not yet been evaluated. See Figure 2. In the largest study in pediatrics, of 300 attempts, 294 TNEs were successfully performed (98% success rate) in 190 children and young adults, with ages ranging from 3 to 22 years (31). The biopsy specimens obtained by TNE were all adequate for assessment of EoE (30, 31). There were no major adverse events and TNE reduced costs by over 50% compared with EGD under anesthesia (31). In addition, qualitative studies show that the overall perception and satisfaction of TNE for parents and patients was positive (30, 32). Unsedated TNE has advantages because it can be performed in an outpatient clinic room and reduces the risk and cost associated with anesthesia. This is particularly relevant to children with EoE, who often require serial endoscopy.

### Endoscopic Functional Lumen Imaging Probe

Endoscopic functional lumen imaging probe (FLIP) is a novel endoscopic assessment tool to measure caliber and distensibility of the esophageal lumen. It uses impedance planimetry during volume-controlled distention of the esophagus to provide measurements of pressure and dimensions of the esophagus and gastroesophageal junction (33). Studies in adults and children have shown that esophageal distensibility is decreased in patients with EoE compared to non-EoE controls. Lower distensibility is associated with the occurrence of food impaction and the need for dilation, important patient outcomes in EoE, and distensibility has been shown to improve with treatment (34–37). In children and adolescents, lower distensibility was associated with active inflammation as compared to the distensibility in patients with inactive/treated EoE (36). FLIP is likely to be a useful and practical tool in the assessment of disease severity and disease phenotype assessment.

### Less Invasive Methods to Assess Disease Activity

EoE management often requires frequent assessment of histologic changes in response to therapeutic adjustments particularly in the case of dietary management of EoE. TNE obviates the need for anesthesia but still allows for endoscopic and histologic assessment. Less invasive means of sampling the esophageal lumen without endoscopy are being developed and may eventually alter the way in which we assess disease response. Developed sampling methods include the cytosponge and the esophageal string test. The cytosponge consists of an ingestible gelatin capsule containing a compressed mesh sponge attached to a string developed initially for esophageal cancer screening. As the sponge passes back up through the esophagus, a tissue specimen is collected to create a tissue pellet that can then be evaluated for histologic assessment. In an adult study, eosinophil counts highly correlated between the biopsy and cytosponge (38). At the time of this writing, the cytosponge has yet to be studied in children or adolescents. Given the size of the mesh sponge, it may have limited use in pediatrics or in patients with esophageal narrowing.

The esophageal string test (EST) similarly calls for swallowing a gelatin capsule. In the EST, a weighted gelatin capsule containing 90 cm of nylon string is swallowed while one end is taped to the side of the face. The esophageal portion is analyzed for the presence of eotaxin-3 and eosinophil major basic protein-1 and an EoE score resulted. Combined, these two biomarkers strongly associated with eosinophil density and had AUC 0.86 for identifying active EoE (39).

Other technologies, such as mucosal impedance, have been studied in the assessment of mucosal inflammation in EoE. Real-time mucosal impedance measurements correlate with esophageal eosinophilia and treatment improves mucosal impedance (40–42). Mucosal impedance probes are placed at the time of endoscopy. As these and other techniques show promise in the research setting, clinical need will encourage the incorporation of these technologies in devices that do not require sedated endoscopy. The EST or other biomarkers are unlikely to take the place of initial diagnostic endoscopy, however less invasive means of sampling the esophageal lumen may allow for less burdensome longitudinal assessment in the management of this chronic disease; hopefully leading to fewer endoscopies for patients without sacrificing control of inflammation.

## Conclusion

Endoscopy is essential to the diagnosis and management of EoE including the attainment of mucosal biopsies, visual inspection of the esophagus and, when needed, therapeutic intervention. Newer endoscopic tools such as FLIP allow for measurement of esophageal distensibility and esophageal remodeling that occurs in EoE. This can provide a complementary assessment of the esophagus together with mucosal inflammation and endoscopic appearance. TNE and novel less or non-invasive means of sampling the esophageal mucosa and/or lumen aim to lessen the burden of repeated endoscopy in this population. Ideally these tools will be able to provide practical assessment of disease activity in the longitudinal management of patients.

## Data Availability Statement

The original contributions presented in the study are included in the article/supplementary material, further inquiries can be directed to the corresponding author/s.

## Author Contributions

NN, RK, and CM-K contributed to the concept development, writing and review of this manuscript, and provided final approval of the version to be published. All authors contributed to the article and approved the submitted version.

## Conflict of Interest

The authors declare that the research was conducted in the absence of any commercial or financial relationships that could be construed as a potential conflict of interest.

## Publisher's Note

All claims expressed in this article are solely those of the authors and do not necessarily represent those of their affiliated organizations, or those of the publisher, the editors and the reviewers. Any product that may be evaluated in this article, or claim that may be made by its manufacturer, is not guaranteed or endorsed by the publisher.
